# Quantitative patterns between plant volatile emissions induced by biotic stresses and the degree of damage

**DOI:** 10.3389/fpls.2013.00262

**Published:** 2013-07-23

**Authors:** Ülo Niinemets, Astrid Kännaste, Lucian Copolovici

**Affiliations:** ^1^Estonian University of Life SciencesTartu, Estonia; ^2^Institute of Technical and Natural Sciences Research-Development, Aurel Vlaicu UniversityArad, Romania

**Keywords:** biotic stress, green leaf volatiles, fungal infection, herbivory, quantitative stress dose–response relationships, volatile organic compounds

## Abstract

Plants have to cope with a plethora of biotic stresses such as herbivory and pathogen attacks throughout their life cycle. The biotic stresses typically trigger rapid emissions of volatile products of lipoxygenase (LOX) pathway (LOX products: various C_6_ aldehydes, alcohols, and derivatives, also called green leaf volatiles) associated with oxidative burst. Further a variety of defense pathways is activated, leading to induction of synthesis and emission of a complex blend of volatiles, often including methyl salicylate, indole, mono-, homo-, and sesquiterpenes. The airborne volatiles are involved in systemic responses leading to elicitation of emissions from non-damaged plant parts. For several abiotic stresses, it has been demonstrated that volatile emissions are quantitatively related to the stress dose. The biotic impacts under natural conditions vary in severity from mild to severe, but it is unclear whether volatile emissions also scale with the severity of biotic stresses in a dose-dependent manner. Furthermore, biotic impacts are typically recurrent, but it is poorly understood how direct stress-triggered and systemic emission responses are silenced during periods intervening sequential stress events. Here we review the information on induced emissions elicited in response to biotic attacks, and argue that biotic stress severity vs. emission rate relationships should follow principally the same dose–response relationships as previously demonstrated for different abiotic stresses. Analysis of several case studies investigating the elicitation of emissions in response to chewing herbivores, aphids, rust fungi, powdery mildew, and *Botrytis*, suggests that induced emissions do respond to stress severity in dose-dependent manner. Bi-phasic emission kinetics of several induced volatiles have been demonstrated in these experiments, suggesting that next to immediate stress-triggered emissions, biotic stress elicited emissions typically have a secondary induction response, possibly reflecting a systemic response. The dose–response relationships can also vary in dependence on plant genotype, herbivore feeding behavior, and plant pre-stress physiological status. Overall, the evidence suggests that there are quantitative relationships between the biotic stress severity and induced volatile emissions. These relationships constitute an encouraging platform to develop quantitative plant stress response models.

## INTRODUCTION

**FIGURE 1:**
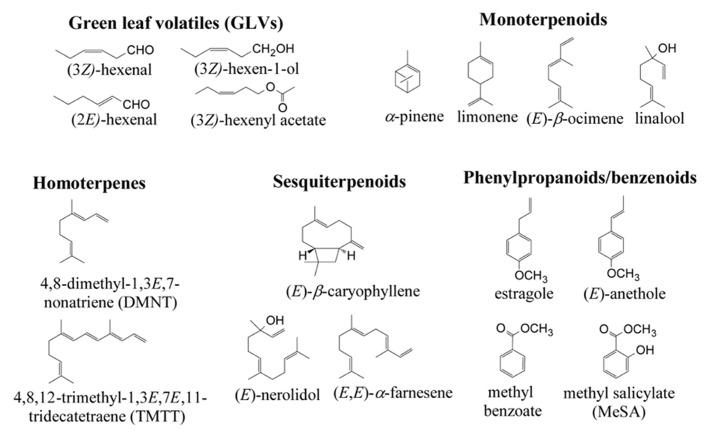
**Molecular structures of selected plant volatiles (BVOC) emitted in response to a variety of stress factors.** Green leaf volatiles (GLV), also called volatiles of lipoxygenase pathway (LOX) are formed via the lipoxygenase pathway and constitute the ubiquitous stress response (Hatanaka et al., 1978; Hatanaka, 1993; Howe and Schaller, 2008). Terpenoids comprise the largest class of plant secondary metabolites. Various terpenoids are emitted in several constitutive emitters, and emissions of specific terpenoids are elicited in response to different stresses (Degenhardt et al., 2009; Llusià et al., 2010, 2013; Loreto and Schnitzler, 2010). Emissions of benzenoids and phenylpropanoids have been less investigated, but the constitutive emissions of these compounds are often characteristic to flowers, and sometimes to leaves (Gang et al., 2001; Dudareva et al., 2004; Yang et al., 2009; Zhao et al., 2010). Furthermore, methyl salicylate is a characteristic stress-induced volatile (Karl et al., 2008; Zhao et al., 2010), and there is evidence that methyl benzoate may also be released in response to stress (Zhao et al., 2010). Different biochemical pathways are responsible for synthesis of different compound classes (**Figure 2**).

**FIGURE 2:**
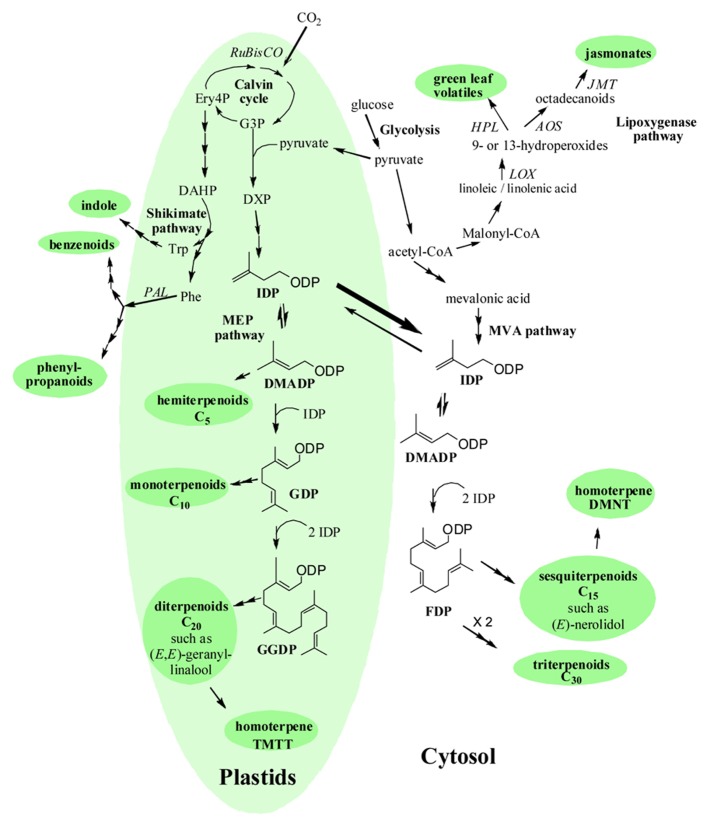
**Simplified scheme of the interactions among the biosynthetic pathways responsible for volatile and non-volatile stress metabolites in plants.** Pathway names are in italics, volatile compound classes are in bold font inside ellipses, and the key enzymes involved in the biosynthetic pathways are next to the arrows in italics. Abbreviations: acetyl-CoA, acetyl coenzyme A; *AOS*, allene oxide synthase; DAHP, 3-deoxy-D-arabino-heptulosonate 7-phosphate; DMADP, dimethylallyl diphosphate; DMNT, 4,8-dimethyl-1,3*E*,7-nonatriene; DXP, 1-deoxy-D-xylulose 5-phosphate; Ery4P, erythrose 4-phosphate; F6P, fructose 6-phosphate; FDP, farnesyl diphosphate; G3P, glyceraldehyde-3-phosphate; GGDP, geranylgeranyl diphosphate; GDP, geranyl diphosphate; HPL, fatty acid hydroperoxide lyases; IDP, isopentenyl diphosphate; JMT, jasmonic acid carboxyl methyl transferase; LOX, lipoxygenase; MEP-pathway, methylerythritol 4-phosphate pathway; MVA, mevalonic acid; PAL, phenylalanine ammonia lyase; PEP, phosphoenolpyruvate; Phe, phenylalanine; TMTT, 4,8,12-trimethyl-1,3(*E*),7(*E*),11-tridecatetraene. The lipoxygenase pathway starts with the dehydrogenation of linolenic and linoleic acids at C_9_ or C_13_ position by lipoxygenases forming 9-hydroperoxy and 13-hydroperoxy derivates of polyenic acids (Hatanaka, 1993; Howe and Schaller, 2008). These compounds are further cleaved by hydroperoxide lyases into oxoacids and C_6_-aldehydes. These aldehydes can be converted into the corresponding alcohols by alcohol dehydrogenases (Hatanaka et al., 1978; Hatanaka, 1993; Dudareva et al., 2006). Terpenoids originate from isopentenyl diphosphate (IDP) and dimethylallyl diphosphate (DMDP), which are synthesized via two different pathways. The cytosolic mevalonate (MVA) pathway starts with the formation of acetoacetyl-CoA (Dewick, 1999), while the plastidial 2-C-methyl-D-erythritol 4-phosphate/1-deoxy-D-xylulose 5-phosphate pathway (MEP/DOXP pathway) starts with condensation of pyruvate and glyceraldehyde-3-phosphate (Lichtenthaler et al., 1997; Rohmer, 1999). The route in plastids provides precursors for the biosynthesis of isoprene, mono-, and diterpenes, while the cytosol-localized pathway for sesqui- and triterpenes. Precursors of terpenes have been experimentally demonstrated to be transported from plastids to the cytosol (Dudareva et al., 2005; Bartram et al., 2006), referred to as the “cross-talk” between the MEP- and MVA-pathways (for recent reviews on terpenoid synthesis see Li and Sharkey, 2013; Rajabi Memari et al., 2013; Rosenkranz and Schnitzler, 2013). Aromatic volatiles are formed via the shikimic acid pathway, starting by condensation of erythrose 4-phosphate and PEP. After numerous steps via 3-dehydroshikimic acid and chorismate, phenylalanine (Phe) is produced. Phe is further converted to *trans*-cinnamic acid by phenylalanine ammonia lyase. *Trans*-cinnamic acid is a starting point for the synthesis of phenylpropanoids, (e.g., phenylethanol, phenylethylbenzoate) and benzenoids (benzaldehyde, methyl benzoate, methyl salicylate etc.; Boatright et al., 2004; Dudareva et al., 2006). Additionally, tryptophan (Trp), which is the precursor of volatile indole, is biosynthesized via shikimic acid pathway (Paré and Tumlinson, 1996) in chloroplast, while indole itself is synthesized in cytosol (Zhang et al., 2008).

Plants as sedentary organisms cannot escape from attackers and stressors and have to adjust to surrounding environment and biotic attacks through their life cycle. During evolution, plants have evolved various defense strategies, including release of volatile organic compounds (VOCs) from their above-ground organs (Zhang et al., 1999; Zhang and Schlyter, 2004; Huang et al., 2012; Fineschi et al., 2013) into the ambient atmosphere, and even from roots into the soil air space and water (Hiltpold et al., 2011; Turlings et al., 2012). Numerous VOCs have been described, which nevertheless belong to a few broad compound classes, including volatile isoprenoids, volatile products of shikimic acid pathway (phenylpropanoids, benzenoids, indole), carbohydrate and fatty acid cleavage products (**Figure 1** for some examples of characteristic volatiles released from plants and **Figure 2** for their biosynthetic pathways (Knudsen et al., 1993; Dudareva et al., 2006; Qualley and Dudareva, 2008; Dicke and Baldwin, 2010; Fineschi et al., 2013). In a few cases, specialized volatiles such as sulfur-containing glucosinolate cleavage products in Brassicales and Malpighiales and furanocoumarins and their derivatives in Apiales, Asterales, Fabales, Rosales, and Sapindales are produced (Berenbaum and Zangerl, 2008; Agrawal, 2011).

In most cases, plant odors are complex mixtures of compounds, reflecting upregulation of multiple pathways, and synthesis of multiple compounds within given pathway. Due to significant differences in physico-chemical characteristics of VOCs within and among the different compound classes (Niinemets et al., 2004), the release kinetics, compound life-time in the ambient atmosphere and uptake by neighboring vegetation strongly vary (Baldwin et al., 2006; Arneth and Niinemets, 2010; Holopainen et al., 2013).

Plant volatile emissions can be constitutive or they can be induced in response to a variety of stresses. Independent of the way of emission, airborne volatiles are thought to be involved in defense reactions elicited by herbivores, pathogens, and even against abiotic stress factors (Dicke and Baldwin, 2010; Fineschi et al., 2013; Possell and Loreto, 2013). These defense responses can be either direct or indirect. In the case of direct responses, emitted volatiles themselves participate in defense or in stress tolerance (Martin and Bohlmann, 2005; Vickers et al., 2009). In the case of indirect defenses, volatiles released serve as infochemical signals eliciting systemic responses within the plant and/or neighboring plants and/or they serves as cues attracting enemies of herbivores (Dicke et al., 1999; Halitschke et al., 2000; Dicke and Bruin, 2001; Heil and Kost, 2006; Heil and Silva Bueno, 2007).

Induced plant defense triggered by plant hormones (Fäldt et al., 2003; Kappers et al., 2010), herbivores (Arimura et al., 2005, 2011), or pathogen attacks (Jansen et al., 2009; Kant et al., 2009; Toome et al., 2010) has been extensively studied. However, much of the work on plant defense responses, in particular on biotic stress responses, has been non-quantitative. Stress-driven plant VOC emission responses have rarely been characterized in relation to the severity of the stress. Yet, this is relevant because mild vs. severe stress might qualitatively alter plant response, either leading to stress priming and adaptation or to hypersensitive response (Heil and Kost, 2006; Frost et al., 2008a; Niinemets, 2010). Quantitative patterns among stress severity and VOC release have been demonstrated for several abiotic stresses including ozone stress (Beauchamp et al., 2005), heat (Karl et al., 2008; Copolovici et al., 2012) and frost stress (Copolovici et al., 2012) and stress induced by diffusely dispersed environmental pollutants such as textile colorants (Copaciu et al., 2013) and antibiotics’ residues (Opricş, 2013).

Studies of VOC emissions triggered by biotic stresses have been mostly investigated qualitatively (but see e.g., Gouinguené et al., 2003; Schmelz et al., 2003a, b; Copolovici et al., 2011). This reflects the focus of plant–herbivore interactions research on overall stress patterns elicited by severe or moderately severe stress. This research has often been driven by the question of how the elicited compounds participate in communication at different trophic levels. In studies focused on plant responses, lack of quantitative investigations might be related to difficulties in characterizing the severity of biotic stress, and to presence of multiple confounding effects that can result from genotypic differences, plant physiological status, and interactions with environmental drivers. As in the nature plants are under continuous pressure of biotic stresses of differing severity, we argue that the overall lack of quantitative stress dose vs. plant response studies is an important shortcoming. Without knowing the stress dose vs. plant response patterns, plant stress responses in the field under strongly fluctuating stress levels cannot be predicted.

In this paper, we first analyze general patterns of constitutive and induced emissions to clearly define what we consider as an induced emission response and analyze how both types of emissions can benefit plants. Then we analyze mechanisms of immediate stress-elicited emissions and systemic responses, review several case studies asking whether biotic stress severity and plant volatile emission responses are quantitatively related, and finally consider some of the difficulties complicating interpretation of stress severity vs. plant emission responses.

## ROLE OF CONSTITUTIVE EMISSIONS OF VOLATILE ORGANIC COMPOUNDS IN PLANT DEFENSE REACTIONS

Constitutive emissions are present in all species storing volatiles in specialized storage tissues, e.g., resin ducts in conifers and glandular trichomes in Labiatae. There is continuous release of these volatiles from the storage structures determined by the rate of compound diffusion, and thus, mainly driven by the compound volatility and temperature (for recent reviews see Monson et al., 2012; Grote et al., 2013). In addition, several widespread species lacking specialized storage compartments synthesize volatile isoprenoids, in particular, isoprene or/and monoterpenes, in light- and temperature-dependent manner (for reviews see Grote et al., 2013; Li and Sharkey, 2013; Monson, 2013).

Constitutive emissions occur both during periods when plants do not experience stress, and when they do. However, emission rates of constitutively released compounds can acclimate to variations in environmental conditions, in particular, to average light and temperature, over days to weeks preceding the sampling (Niinemets et al., 2010a; Monson, 2013). Environmental and biotic stress can also alter the rate of constitutive emissions, either increasing or reducing the emission rates depending on stress severity and duration, and plant ontogenetic status (Niinemets et al., 2010a; Monson, 2013; Possell and Loreto, 2013). In the following, we analyze the ways by which constitutive emissions can increase plant resistance to environmental and biotic stresses.

### PROTECTION BY NON-STORED VOLATILES

Non-stored constitutively released volatiles can directly participate in abiotic defenses by stabilizing membranes and serving as antioxidants (Sharkey et al., 2008; Chen et al., 2009; Vickers et al., 2009; Possell and Loreto, 2013). The synthesized volatiles partition to leaf liquid and lipid phases according to their equilibrium partition coefficients (Niinemets and Reichstein, 2002; Niinemets et al., 2004; Niinemets et al., 2010b). Lipid solubilization of hydrophobic volatiles possibly enhances lipid–lipid and lipid–protein interactions in membranes at higher temperatures (Sharkey et al., 2008; Vickers et al., 2009; Possell and Loreto, 2013), thereby increasing plant tolerance to elevated temperatures (Sharkey and Singsaas, 1995; Loreto et al., 1998; Copolovici et al., 2005). Enhancement of thermal tolerance has been first demonstrated for isoprene (Sharkey and Singsaas, 1995; Singsaas et al., 1997) and then for monoterpenes (Loreto et al., 1998; Copolovici et al., 2005; Llusià et al., 2005). However, not all monoterpenes appear to be equally effective (Copolovici et al., 2005).

Due to their antioxidative characteristics, solubilized volatiles can also quench stress-generated reactive oxygen species (ROS), production of which becomes enhanced during thermal stress, but also during many other abiotic stresses such as ozone stress (Sharkey et al., 2008; Vickers et al., 2009; Possell and Loreto, 2013). The protective effect of volatile isoprenoids can be particularly relevant under drought when stomata close, resulting in elevated leaf temperatures due to reduced transpiratory cooling of leaves. These are also the conditions that lead to a major buildup of volatiles inside the leaves (Sharkey and Singsaas, 1995; Singsaas et al., 1997).

Apart from involvement in abiotic stress tolerance, constitutively released non-stored volatiles can play an important role in host plant selection by herbivores as well as possible deterrents for herbivores (Zhang et al., 1999; Pichersky and Gershenzon, 2002; Degenhardt et al., 2003; Xugen and Luqin, 2006; Loivamäki et al., 2008; Brilli et al., 2009).

### DEFENSES CONFERRED BY STORED VOLATILES

Due to their toxicity, release of compounds stored in specialized storage compartments is known to deter and reduce the feeding activity of herbivores and inhibit biological activity of pathogens (Popp et al., 1995; Ward et al., 1997; Litvak and Monson, 1998; Baier et al., 2002). The emissions of stored volatiles may also serve as important signals in host plant selection (Kelsey and Joseph, 1997; Mita et al., 2002). Involvement of constitutive storage emissions in protecting from abiotic stresses has not been demonstrated, although due to continuous emission, a certain, relatively high, vapor pressure of storage volatiles is maintained in leaf intercellular air space. Depending on compound physico-chemical characteristics (Niinemets et al., 2004; Harley, 2013), the vapor pressure supported by storage emissions can result in equilibrium compound concentrations in leaf liquid and lipid phases that are comparable to those observed for non-storage emissions of isoprene and monoterpenes. This suggests that emissions from storage structures can fulfill analogous functions in abiotic stress tolerance as the constitutive emissions in species lacking the storage.

## INDUCED VOLATILES IN PLANT DEFENSE RESPONSES: FROM QUALITATIVE TO QUANTITATIVE PATTERNS

While only certain plant species are constitutive emitters, all plant species typically respond to stress by triggering emissions of a variety of characteristic stress volatiles (**Figure 2**, Paré and Tumlinson, 1999; Kessler and Baldwin, 2001; Loreto and Schnitzler, 2010; Niinemets, 2010). Here we briefly consider what are induced emissions, what is emitted, what is the biological role of induced emissions, and by which mechanisms induced emissions could be coupled to stress severity in a dose-dependent manner.

### DEFINITION OF INDUCED EMISSIONS

Differently from constitutive emissions, emissions of stress volatiles during periods intervening stress events are only present at very low background levels, often close to the detection limit of analytical systems (e.g., Toome et al., 2010; Copolovici et al., 2011, 2012). Stress leads to amplification of these emissions by several orders of magnitude (Turlings et al., 2004), and after stress relief, the emissions again decrease to the background level (Copolovici et al., 2011; Karban, 2011). Yet, relaxation of emissions after stress typically takes longer than elicitation (Degenhardt and Lincoln, 2006; Karban, 2011). The main difference among constitutive and induced emissions is not whether or whether not different types of emissions respond to stress. Both types of emissions are stress-responsive, but the stress sensitivity of constitutive and induced emissions is very different, and the level of emission under non-stressed conditions is also different. Detectable induced emissions are only present during stress and during the relaxation period after stress.

We emphasize that induced emissions are generally understood as stress-driven emissions of *de novo* synthesized volatiles (Paré and Tumlinson, 1997; Niinemets et al., 2010b). In constitutively emitting species storing volatiles in specialized compartments, wounding due to herbivory may break the storage compartments, resulting in emission bursts of the stored compounds (e.g., Loreto et al., 2000; Danielsson et al., 2008). Strictly speaking, these emissions should not be called “induced” emissions as wounding results in major enhancement of diffusion of already synthesized compounds rather than a physiological response.

### WHAT COMPOUNDS ARE INDUCED?

Various compound classes are induced with differing kinetics, reflecting different emission mechanisms. Induced emissions of some compounds such as green leaf volatiles (volatile products of lipoxygenase (LOX) reaction, LOX products), are emitted within minutes after the start of stress (e.g., Loreto et al., 2006), and reflect activation of already available enzymatic apparatus. In the case of LOX volatiles, rapid emissions are triggered by the release of free fatty acids from cell membranes, and their peroxidation by LOX enzymes (Feussner and Wasternack, 2002; Liavonchanka and Feussner, 2006). In addition, initial stress responses lead to activation of a large number of genes, several of which are responsible for the biosynthesis of defensive plant volatiles, including characteristic monoterpenoids, sesquiterpenoids, and homoterpenes. As the result, emissions of these stress volatiles are induced in hours to days after the start of the sustained stress or following a single stress event (Beauchamp et al., 2005; Copolovici and Niinemets, 2010; Copolovici et al., 2011). (*E*)-β-Ocimene, linalool, methyl salicylate (MeSA), indole, (*E*, *E*)-α-farnesene, (*E*)-β-farnesene and homoterpenes 4,8-dimethyl-1,3*E*,7-nonatriene (DMNT) and 4,8,12-trimethyl-1,3(*E*),7(*E*),11-tridecatetraene (TMTT) are characteristic stress compounds in various plant species (**Figure 1**; Paré and Tumlinson, 1999; Frey et al., 2000; Vuorinen et al., 2007; Toome et al., 2010; Copolovici et al., 2011, 2012; Zhuang et al., 2012). For example in our previous studies, next to LOXpathway products (*Z*)-3-hexenol, (*E*)-2-hexenal, 1-hexanol, and (*Z*)-3-hexenyl acetate, feeding of foliage of temperate deciduous tree *Alnus glutinosa* by larvae of the geometrid moth *Cabera pusaria* induced also the emission of a homoterpene DMNT and a sesquiterpene (*E*, *E*)-α-farnesene (Copolovici et al., 2011), which are not released by the foliage of non-stressed *Alnus glutinosa* (Lindfors et al., 2000).

### INFORMATION CARRIED BY INDUCED VOLATILES

**FIGURE 3:**
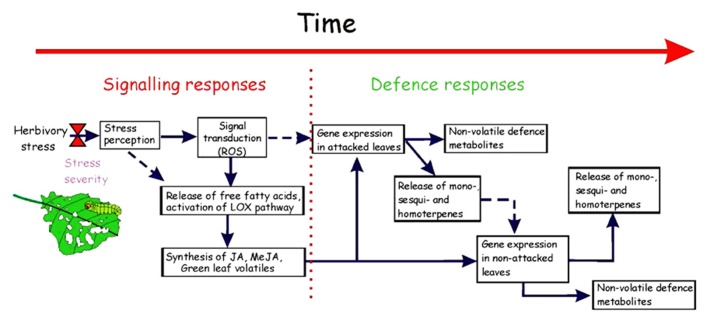
**Flow path of time-dependent herbivory-driven signaling and defense responses.** Herbivory damage leads to a rapid, within minutes, oxidative burst and release of free fatty acids from plant membranes in the immediate location of damage (Feussner and Wasternack, 2002; Maffei et al., 2007; Arimura et al., 2011; Spinelli et al., 2011). This leads to activation of lipoxygenase pathway that results in release of green leaf volatiles (a variety of C_6_ aldehydes) and synthesis of jasmonate and methyl jasmonate (Feussner and Wasternack, 2002). Sometimes, depending on attacking organism, the early signaling responses also include ethylene and methyl salicylate (Maffei et al., 2007; Mithöfer and Boland, 2008; Arimura et al., 2011). Further cascade of events includes activation of defense gene expression that leads to synthesis of a variety of volatile isoprenoids and also production of non-volatile defense compounds such as polyphenols. Gene expression patterns may be directly elicited by reactive oxygen species (ROS) dependent activation of MAP kinases, but these responses more commonly include activation of hormonal pathways (Arimura et al., 2011). Volatile and non-volatile phytohormones released by attacked leaves can elicit defense gene expression in non-attacked leaves (systemic response; Maffei et al., 2007; Mithöfer and Boland, 2008; Arimura et al., 2011). Uncertain or less frequent paths are shown by dashed arrows.

Timing, amount, and composition of stress-triggered emissions can carry information about the emitting species, and type of the stress, while timing and amount can reflect the severity of the stress (Llusià et al., 2002; Beauchamp et al., 2005; Niinemets, 2010; Joó et al., 2011; Copolovici et al., 2012; Fatouros et al., 2012). Thus, induced emissions are thought to serve primarily as infochemicals. LOX-compounds released rapidly after stress are known to serve as “messenger-compounds” in plant–plant communication (Shulaev et al., 1997; Arimura et al., 2001; Farag and Paré, 2002) or in triggering systemic response (Farag and Paré, 2002; Park et al., 2007) that can result in triggering volatile emissions in non-stressed leaves of the same plant and in neighboring plants (**Figure 3**, Röse et al., 1996; Halitschke et al., 2000; Heil and Silva Bueno, 2007; Staudt and Lhoutellier, 2007; Dicke and Baldwin, 2010; Peng et al., 2011). Other rapidly elicited volatiles can also potentially serve as messengers (Paré et al., 2005). These primary airborne messengers can further elicit secondary “messengers” such as jasmonic acid or salicylic acid migrating in liquid phase through phloem to the tissues distant from the stressed ones activating defense genes (Park et al., 2007). For example, in lima bean (*Phaseolus lunatus*) plants infested by spider mites (*Tetranychus urticae*), released volatiles activated multifunctional signaling cascades involving the ethylene and jasmonic acid signaling (Arimura et al., 2002).

The question, however, is how informative are the LOX product emissions triggered by various stresses. Plants have multiple LOXs and fatty acid hydroperoxide lyases (Feussner and Wasternack, 2002), and once synthesized, C_6_ aldehydes can further be chemically modified resulting in formation of alcohols and esters, collectively generating variations in the emission profiles. The composition of emitted LOX products can be similar for different stresses such as caused by mechanical wounding, herbivory, heat, and frost stress even for different species (Brilli et al., 2011; Copolovici et al., 2011, 2012). Yet, it has been recently demonstrated that herbivores can isomerize LOX products, resulting in different emission blends for mechanical wounding and herbivory and altered attractiveness to predators (Allmann and Baldwin, 2010).

While there is broad evidence of convergent responses for different stresses, in reed (*Phragmites australis*), it was demonstrated that heat stress resulted only in the release of (*E*)-2-hexenal, while wounding caused emissions of (Z)-3-hexenal, (*Z*)-3-hexenol, and (*E*)-2-hexenol (Loreto et al., 2006). Moreover, Bai et al. (2011) demonstrated that even chilling or heating can activate LOX pathway differently. The generality of such changes in emission profiles, and the capacity of different LOX volatiles in eliciting systemic responses clearly need further experimental work.

### INDUCED EMISSIONS AS DIRECT DEFENSES

Emissions of isoprenoids triggered in response to stresses are chemically similar to volatiles released in constitutive emitters and could potentially also be involved in direct defense, e.g., serving as antioxidants quenching the ROS formed in plants under stress. Given that the induced emissions are at maximum level when the stress and ROS formation are the greatest, involvement of induced isoprenoids in direct defense is plausible. Such a role could be particularly relevant for the emissions induced by abiotic stresses that likely play a less prominent role in multitrophic signaling. There is currently no evidence of the involvement of induced emissions in direct defense against abiotic stresses, although such a role would be compatible with stress dose-dependent emissions of induced volatiles. However, induced emissions have been demonstrated to serve as repellents of herbivores (Bernasconi et al., 1998).

### MECHANISMS BY WHICH PLANT DEFENSE RESPONSES CAN BE QUANTITATIVELY MODULATED BY STRESS SEVERITY

The sequence of events leading from initial stress response to release of early stress volatiles, activation of gene responses and specific secondary metabolic pathways, and ultimately to elicitation of emissions of “late” stress-specific volatiles and systemic responses has been studied intensively (Byers et al., 2000; Arimura et al., 2005, 2011; Dudareva et al., 2006; Kant et al., 2009). Nevertheless, there are still significant uncertainties in how the stress signal is received, transduced, and amplified (Niinemets, 2010). While abiotic stresses typically impact the entire plant, the entire organ or multiple organs, biotic stress is characteristically more localized. For instance, depending on species, chewing herbivores start feeding at the margins or form perforations and skeletonized spots within the lamina, while sap-sucking insects typically attack the phloem in the veins. The spread of the damage from the initial localized damage site(s) increases during the course of feeding and depends on the number of insects attacking simultaneously the leaf. Analogously, in plant–pathogen interactions, pathogen spores dispersed by water, wind, or by insects settle on a plant and form hydrophobic interactions with the waxy polymers on leaf surface. Ultimately, the airborne pathogen enters the leaf intracellular space via stomata (El Omari et al., 2001; Prats et al., 2007). The density of pathogen propagules determines the number of stomatal entry points within the given leaf, but the initial response remains characteristically localized unless the pathogen density is very high. Thus, in the case of biotic stresses, the stress severity often increases in time and in spatial coverage.

The key question is how the initial stress localized in the impacted area of the leaf is sensed by the plant and to what extent the stress response is affecting neighboring non-impacted areas and surrounding non-impacted leaves. The other important question is how the overall stress response is associated with the total impacted area (stress dose). In the case of herbivory by chewing insects, chewing damage, i.e., rupture of cell walls, breakage of cellular membranes, and exposure of cell contents to ambient environment, itself can elicit activation of LOX pathway and release of LOX volatiles that can serve as signals for subsequent stress responses (**Figure 3**, Maffei et al., 2007; Howe and Schaller, 2008; Mithöfer and Boland, 2008). There is also evidence that insect-driven elicitors such as β-glucosidase (Mattiacci et al., 1995) or fatty-acid conjugate such as volicitin (Alborn et al., 1997) from the oral secretion of herbivores are triggering the early stress response after becoming in contact with the wounded plant tissue. Such an early stress response includes membrane depolarization, and increases of cytosolic Ca^2^^+^ level (Dombrowski and Bergey, 2007) that activate calmodulin and other Ca^2^^+^-sensing proteins such as mitogen-activated protein kinase (MAPK) pathways (Nakagami et al., 2005; Maffei et al., 2006, 2007; Howe and Schaller, 2008; Mithöfer and Boland, 2008; Vadassery et al., 2012). Localized generation of ROS, including superoxide ($O_{2}^{-}$), hydrogen peroxide (H_2_O_2_), and hydroxyl radicals (HO^•^ Foyer and Noctor, 2003), is further involved in regulating plant defense reactions, including activation of MAPK pathways, and elicitation of jasmonic acid or salicylic acid-dependent signaling and gene expression (Desikan et al., 2001; Maffei et al., 2007). It is at this point the LOX pathway is activated leading to emission of LOX products (Maffei et al., 2007).

The non-volatile signal molecules may move from the site of immediate damage to other parts of the leaf and plant through plant apoplast (cellular water and xylem), and through cytosolic path in plasmodesmata and phloem. Furthermore, hypersensitive response in the case of some biotic interactions can “seal off” the damaged area (Lam et al., 2001; Yoda et al., 2003), reducing the propagation of the signal. Volatile airborne signals are more efficiently transmitted over longer distances (Heil and Ton, 2008), but their formation likely requires physical presence of the stressor(s) at the impact sites. In fact, when the stress is relieved, the signal propagation and defense response is silenced as evidenced by reduced activity of expression of defense genes (Hundertmark and Hincha, 2008). Although the expression level of some defense genes may remain high after the stress indicating stress priming (Bruce et al., 2007; Hundertmark and Hincha, 2008), stress-triggered volatile emissions also decrease to the low background level that was observed before the stress (Copolovici et al., 2011). Thus, in the case of biotic stresses, continuous elicitation may be needed to keep the stress-dependent pathways active and maintain induced volatile emissions at high rates. On the other hand, this suggests that more simultaneous sites of damage may be associated with greater emission rates of volatiles, resulting in quantitative stress dose vs. emission relationships. However, this simplified mechanism has difficulties in scaling from localized responses to systemic elicitation of volatile emissions (Röse et al., 1996; Farag and Paré, 2002; Staudt and Lhoutellier, 2007). Furthermore, it is currently unclear how the systemic response is quenched after stress. If sustained systemic elicitation needs a continuous flow of signal molecules from the immediate site of damage, systemic emissions can also depend on the severity of the stress in a dose-dependent manner. It is even plausible that the rate of induction of systemic response depends on the elicitor dose. The higher the concentration of the elicitor in the ambient atmosphere the higher the proportion of elicitor’s binding sites that are filled.

## EVIDENCE OF DOSE–RESPONSE RELATIONSHIPS UNDER BIOTIC STRESSES FROM CASE STUDIES

As discussed above, the emission rates of key induced volatiles, including LOX products and monoterpenes, have been shown to scale quantitatively with the severity of several abiotic stresses. The evidence also suggests that the propagation of damage and the number of simultaneous stress impact sites can also lead to quantitative relationships between the severity of biotic stress and release of induced volatiles. Here we analyze several case studies that suggest that the biotic stresses can elicit volatiles in dose-dependent manner similarly to abiotic stresses.

### HERBIVORY- AND WOUNDING-ELICITED EMISSIONS IN RELATION TO STRESS “SEVERITY”

**FIGURE 4:**
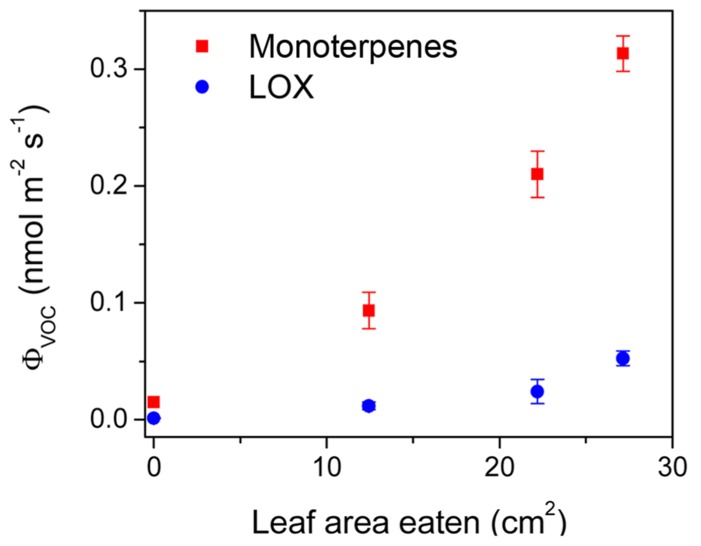
**Emissions of monoterpenes and volatile products of lipoxygenase (LOX) pathway from the leaves of temperate deciduous tree *Alnus glutinosa* in relation to the amount of leaf area eaten by the larvae of the geometrid moth *Cabera pusaria*.** Different amounts of leaf area correspond to different numbers of larvae feeding on the plant (0, 2, 4, 8 larvae). Modified from Copolovici et al. (2011).

Caterpillars feeding on leaves typically damage the plant by chewing or tearing off leaf pieces, thereby eliciting the classic release of LOX volatiles, followed by the emissions of terpenoids and shikimic acid pathway products (e.g., Paré and Tumlinson, 1996; Frey et al., 2000; Kessler and Baldwin, 2001; Vuorinen et al., 2004; D’Alessandro et al., 2006). In line with the general patterns, larvae of the geometrid moth, common white wave (*Cabera pusaria*), larvae feeding on the leaves of temperate deciduous tree *Alnus glutinosa* elicited emissions of LOX volatiles, monoterpenes, sesquiterpenes, and homoterpene DMNT (Copolovici et al., 2011). The emission response depended on the number of larvae feeding simultaneously on the given plant and on the degree of leaf damage (**Figure 4**). In particular, the emission rates of the sum of different LOX volatiles and the sum of monoterpenes and sesquiterpene (*E*, *E*)-α-farnesene were quantitatively associated with the severity of herbivory stress (Copolovici et al., 2011). However, differences in emission rates were smaller for some compounds such as homoterpene DMNT, which seemed to be informative of the presence of herbivores, but these differences were not quantitatively associated with the degree of damage (Copolovici et al., 2011).

There is further quantitative and semi-quantitative evidence of dose-dependent release of stress volatiles from wounding, herbivory, or elicitor treatment studies. Brilli et al. (2011) demonstrated that in the grass *Dactylis glomerata*, the amount of LOX volatiles released after mechanical wounding scaled linearly with the length of excision. In another study with *Alnus glutinosa*, there were quantitative relationships between the number of larvae of green alder sawfly (*Monsoma pulveratum*) and the leaf area damaged and the emission of LOX products and monoterpenes (Copolovici et al., unpublished data 2013). In *Zea mays* fed by *Spodoptera littoralis* larvae, the sum of all volatiles emitted (LOX products, indole and mono-, homo-, and sesquiterpenes) was positively correlated with the number of larvae (0–32) feeding (Turlings et al., 2004). In the same plant–herbivore model system, the sum of volatiles increased curvilinearly with the overall degree of plant damage (Gouinguené et al., 2003). In another study with *Z. mays* treated with various elicitors, sesquiterpene emissions increased curvilinearly with the amount of applied *Spodoptera littoralis* elicitor volicitin as well as with the amount of applied jasmonic acid, and with the concentration of ethylene during fumigation; indole emissions also scaled positively with ethylene concentration (Schmelz et al., 2003b). In a further investigation in *Z. mays* fed by larvae of *Spodoptera exigua* (Schmelz et al., 2003a), emissions of indole and sesquiterpenes were positively correlated with the degree of infestation. The degree of infestation was further associated with greater endogenous jasmonic acid levels and greater ethylene production rate, and overall, there were strong positive curvilinear relationships between endogenous jasmonic acid concentration and the rates of emission of sesquiterpenes and indole (Schmelz et al., 2003a). In *Medicago truncatula* infested with aphid* Acyrthosiphon kondoi*, transcript levels for genes characterizing the activity of salicylic acid-dependent signaling strongly increased with the plant infestation score; to some extent, transcripts for several LOX- and jasmonic acid-dependent signaling pathway enzymes also scaled positively with the extent of infestation (Gao et al., 2008). Although the degree of damage was not quantified, there is further evidence of increased emission of stress volatiles with the spread of infestation in *Brassica oleracea* fed by *Pieris brassicae* larvae (Scascighini et al., 2005) and in *Aesculus hippocastanum* infested by *Cameraria ohridella* larvae (Johne et al., 2006). These studies collectively provide conclusive evidence that the stress-dependent elicitation of emissions is linked to the severity of herbivory and mechanical damage or degree of infestation in a dose-dependent manner.

### QUANTITATIVE RESPONSES TO PATHOGEN ATTACKS

Attacks by pathogenic fungi such as rust fungi, powdery mildews or *Botrytis cinerea* also lead to emissions of LOX volatiles and release of characteristic terpenoids (Heath, 1997; Steindel et al., 2005; Jansen et al., 2009, 2011; Toome et al., 2010). Leaf rust fungi are biotrophic pathogens and need living host tissue for nutrients and carbon. In contrast, powdery mildews and *Botrytis* *cinerea* are necrotrophic fungi, which kill the host tissue and adsorb the carbon and nutrients from the dead cells. Both rust fungi and powdery mildews are highly specialized obligate plant parasites (Staples, 2000; Glawe, 2008; Duplessis et al., 2011), while *Botrytis* *cinerea * is a wide-spectrum plant parasite (Staats et al., 2005)*.*

**FIGURE 5:**
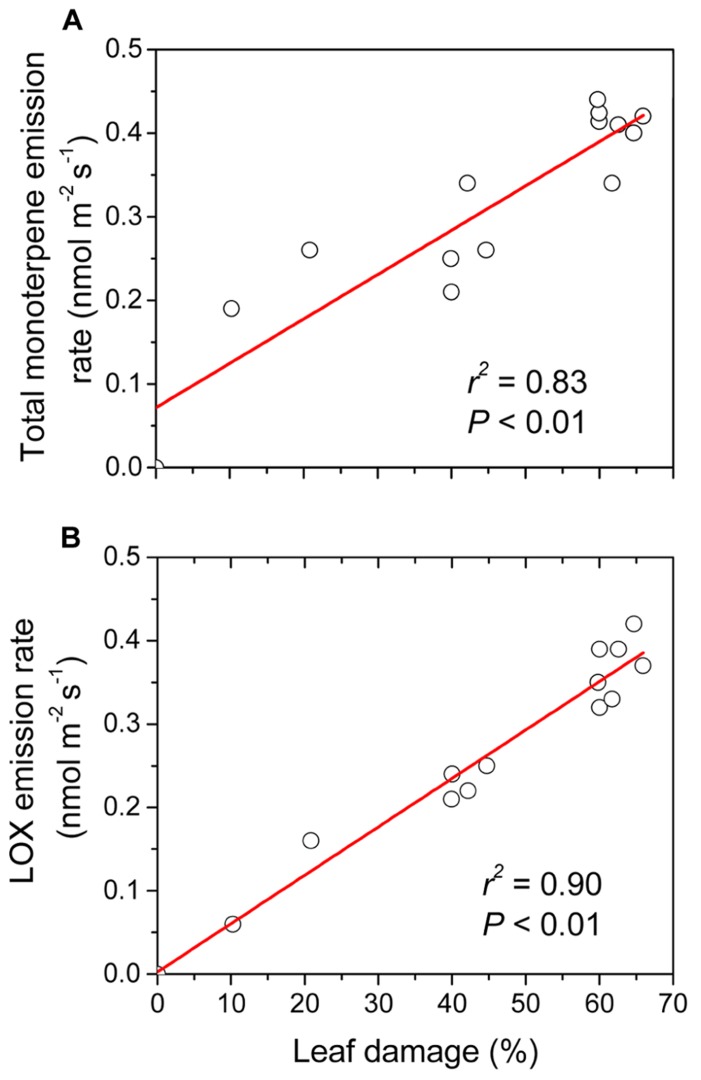
**Emissions of monoterpenes (A) and volatile products of LOX pathway **(B)** from the leaves of temperate deciduous tree *Quercus robur* infected with oak powdery mildew (*Erysiphe alphitoides*) in relation to the percentage of leaf area infected (unpublished data of Copolovici and Niinemets).** Volatile collection and analysis from the infected leaves follows the protocol as described in detail in Copolovici et al. (2009), 2011, (2012).

In the case of rust fungus *Melampsora* infecting hybrid willow (*Salix burjatica *× *S. dasyclados*) foliage, emissions of LOX volatiles, monoterpene (*Z*)-β-ocimene and sesquiterpenes increased with the spread of infection (Toome et al., 2010), indicating that the degree of fungal colonization and volatile emissions were quantitatively related. In the case of oak powdery mildew (*Erysiphe alphitoides*) infecting the leaves of *Quercus robur*, emissions of LOX volatiles and monoterpenes scaled close to linearly with the percentage of leaf area infected with mildew (**Figure 5**). Analogously, Jansen et al. (2009), demonstrated that in tomato (*Solanum lycopersicum*) plants inoculated with *Botrytis cinerea*, the emissions of LOX volatiles and monoterpenes depended on the severity of infection. At larger scale, sesquiterpene emission from Scots pine (*Pinus sylvestris*) correlated with the number of airborne fungal spores incident to vegetation, and the sesquiterpene emissions were suggested to be indicative of plant response to fungal stress (Hakola et al., 2006).

### WHY ARE THERE QUANTITATIVE STRESS DOSE VS. PLANT RESPONSE RELATIONS IN NATURE?

As a whole, the outlined evidence suggests that biotic stress severity and emission response are quantitatively related even for that different stresses as herbivory and fungal pathogen attacks. Thus, the rate of induced volatile emission can constitute a reliable indicator for the severity of biotic stress at any moment of time in the vegetation. However, the question is what can be the biological significance of such quantitative relationships? From insect behavioral studies, there seems to be a widespread consensus that emissions induced by biotic stress primarily serve as qualitative signals. In fact, in laboratory olfactometer experiments (Mumm and Hilker, 2005; Turlings et al., 2005; Fernandes Furtado Michereff et al., 2011), insect performance was only weakly associated with the volatile concentration, if at all. However, under such conditions, there is no relationship or only a weak relationship between volatile concentration and the distance to the “emission source.” In field conditions, volatile concentration strongly decreases with the distance from the emission source, especially in reactive atmospheres were the rate of compound destruction might be high (Holopainen et al., 2013), and thus, a greater emission rate also implies a greater spread of the signal. In the field, insect performance does depend on the distance from the emitting plant, underscoring the importance of concentration gradients (e.g., Karban, 2001; de Bruyne and Baker, 2008). Thus, stronger emissions are potentially associated with attraction of herbivore enemies from a wider distance.

Another important issue is the connection between the strength of the emission response and the spread of systemic response. Systemic induction has been shown to be stronger closer to the biotic stress site and gradually decrease with the distance from the site of damage (Tuomi et al., 1998; Frost et al., 2008b; Karban, 2011). Thus, a stronger induced emission reaction in response to a more severe herbivore attack or pathogen infestation would result in a greater spread of systemic elicitation, thereby contributing to mobilization of plant defenses to a greater degree against a more probable biotic attack. This reasoning suggests that the capacity to respond stronger to more severe stress can importantly enhance plant fitness.

## COMPLICATIONS IN CHARACTERIZING THE DOSE-DEPENDENCIES OF ELICITED EMISSIONS

Although there is encouraging evidence of quantitative relationships among the severity of biotic stress, the stress “dose,” and the emission rate of induced volatiles, the emission time-courses may be complex and the relationships among the stress severity and emission response may vary among genotypes of the given species, among species and depend on the past stress history and other potentially interacting stresses. Effects of interacting and sequential stresses, including stress interactions, stress sequence, and priming have been addressed in several recent reviews (Dicke and Baldwin, 2010; Holopainen and Gershenzon, 2010; Loreto and Schnitzler, 2010; Niinemets, 2010). Here we highlight modifications in emission rates due differences in elicitation time kinetics, plant genotype and due to variations in plant pre-stress physiological status, substrate availability for production of induced volatiles and physico-chemical constraints on the emission of volatiles.

### DIFFERENCES IN ELICITATION TIME KINETICS

Feeding activity of attacking herbivores varies during the day for different herbivores (De Moraes et al., 2001; Fedderwitz et al., 2012; Goodspeed et al., 2012; Jander, 2012). Plant jasmonate-based defense system also has a strong circadian rhythm that can be synchronized with insect circadian behavior (Goodspeed et al., 2012; Jander, 2012). As the result of circadian rhythm of jasmonate-mediated defenses, emission response triggered by given mechanical or herbivory damage or given elicitor treatment can vary depending on the timing of stress event. In addition, the situation can be further complicated by immediate effects of environmental drivers on the rate of induced volatiles. While LOX products are released shortly after damage or elicitor treatments during both the light and dark periods (Arimura et al., 2008), emissions of terpenoids such as (Z)-β-ocimene and linalool are light-dependent (Niinemets et al., 2002; Hansen and Seufert, 2003; Arimura et al., 2008). Thus, in the case of night-time damage, terpenoid emissions are minor during the night period, but jasmonate-dependent defense pathway is activated quickly after damage and transcripts of pertinent terpenoid synthases accumulate during the night (Arimura et al., 2008). As the result, there is a burst of terpenoid emission as soon as the substrate becomes available with the onset of the light period (Arimura et al., 2008). In the case of day-time feeding, the emissions of terpenoids start during the light period as the photosynthetic substrate is available, but emissions are lower than for the night-time damage (Schmelz et al., 2001; Arimura et al., 2008). This reflects the circumstance that accumulation of terpenoid synthase protein is time-consuming and full terpenoid synthesis activity is not reached on the same day of the leaf damage (Arimura et al., 2008).

Bi-phasic emission time-kinetics have also been observed for several volatiles under different biotic stresses. In the case of *Cabera pusaria* caterpillar feeding, (*E*, *E*)-α-farnesene emissions from *Alnus glutinosa* foliage increased bi-phasically during feeding. The emissions were quantitatively related to the degree of damage at the two maxima, but no significant differences among the treatments of varying severity were observed in the intervening period between the two rising phases (Copolovici et al., 2011). In a similar manner, sesquiterpene and (*Z*)-β-ocimene emissions from the rust fungus *Melampsora* infected *Salix burjatica* × *S. dasyclados* (Toome et al., 2010) and LOX product and monoterpene emissions from *Botrytis cinerea* infected *Solanum lycopersicum* (Jansen et al., 2009) increased bi-phasically after infection. Interestingly, in *Salix burjatica* × *S. dasyclados* (Toome et al., 2010) the secondary increase of sesquiterpene emissions was not associated with LOX volatile emissions. It is tempting to speculate that the first peak reflects the immediate signaling response triggered by the biotic elicitor, while the second peak observed in a few days since the initial stress response is indicative of systemic response to airborne volatiles, and may not necessarily originate from the damaged leaf parts. Understanding the bi-phasic nature of the emissions induced by biotic attacks clearly needs further experimental studies independently analyzing the time kinetics of immediate stress-driven and secondary emissions in attacked and non-attacked foliage.

### DOSE-DEPENDENCIES IN RELATION TO PLANT GENOTYPE AND PRE-STRESS PHYSIOLOGICAL STATUS

The situation is further complicated by significant genotypic variations in the level of emissions induced in response to given biotic stress (Degen et al., 2004; Turlings et al., 2005; Degenhardt et al., 2008; de Vos et al., 2008; Wu et al., 2008; Fernandes Furtado Michereff et al., 2011). These variations are not fully understood, but such genotypic differences have been associated with the overall degree of elicitation of defense pathways by given stress (Wu et al., 2008). The genotypic variation in elicited emissions can also be dependent on the constitutive resistance to given stress (Turlings et al., 2005; Fernandes Furtado Michereff et al., 2011), and thus, the degree of damage may vary among genotypes at given stress severity. As the degree of damage has not been routinely reported in studies investigating genotypic differences in stress-elicited induced emissions, further studies are needed to understand whether the observed differences reflect a real variation in plant response or whether they are driven by differences in the degree of damage.

There is ample experimental evidence demonstrating the relevance of pre-stress physiological status in altering the induced emission rates, composition and time kinetics. In *Z. mays*, volicitin-dependent sesquiterpene emissions were much greater under low N nutrition, and the emissions in N-deficient plants were also more sensitive to ethylene (Schmelz et al., 2003b). Overall upregulation in terpenoid synthesis under N-deficiency has also been observed in camphorweed (*Heterotheca subaxillaris*) (Mihaliak and Lincoln, 1989). In contrast, induced emissions were reduced under low nutrient availability in another study with *Z. mays* (Gouinguené and Turlings, 2002). Enhancement of activities of secondary metabolic pathways under limited N have been explained by a variety of hypotheses including “carbon-nutrient balance” (CNB) or “excess carbon” hypotheses, both based on modifications in plant sink–source relations under stress (Bryant et al., 1983; Herms and Mattson, 1992; Peñuelas and Estiarte, 1998). Yet, there is only partial support to these hypotheses (e.g., Llusià et al., 2010; Sardans et al., 2010; Peñuelas et al., 2011; Kännaste et al., 2013) as also the comparisons among different *Z. mays* experiments demonstrate.

Studies on dose–emission relationships should also standardize other environmental drivers. Terpenoid emissions in *Z. mays* elicited by oral secretion of *Spodoptera littoralis* increased with decreasing soil water availability (Gouinguené and Turlings, 2002), and increased curvilinearly with air humidity, light intensity, and temperature (Gouinguené and Turlings, 2002). These environmental responses are analogous to observations in other species (Staudt and Lhoutellier, 2007; Staudt et al., 2010; Staudt and Lhoutellier, 2011), and are consistent with the strong connection of the production of induced terpenoid volatiles and photosynthetic carbon metabolism (see above). In addition, due to high water-solubility of some of the induced compounds such as linalool, methanol and LOX pathway volatiles, variations in stomatal openness during the day and in response to soil drought can directly affect the emissions of water-soluble volatiles (Niinemets et al., 2002; Niinemets et al., 2004; Harley et al., 2007; Harley, 2013). Thus, in assessing the stress dose vs. induced emission responses, it is important to consider the substrate-level and physico-chemical constraints on the rate of induced volatile production and emission.

## CONCLUSION

Plants in natural environments are under fluctuating pressure of various abiotic and biotic stressors. Despite differing elicitation mechanisms, various stresses tend to converge at the level of ROS signaling (Fujita et al., 2006), and different stresses elicit release of the same ubiquitous stress volatiles such as volatiles of the LOX pathway as well as more stress-specific mono- and sesquiterpene blends and shikimic acid pathway products. While the emissions of volatiles have been quantitatively related to the severity of abiotic stresses, biotic stress severity is more difficult to quantify, especially because biotic infections typically do not influence the whole organ or plant such that there are impacted and non-impacted regions within the leaf and among the leaves in the given plant, and attacked and non-attacked plants within the vegetation. Also, stress-triggered emissions from impacted areas typically elicit systemic response and lead to secondary emissions from non-damaged plant parts. On the other hand, after the cessation of biotic stress, emissions come rapidly to background level, indicating response silencing.

The evidence summarized here collectively demonstrates that volatile release from plant foliage is quantitatively related to the severity of herbivory and pathogen stresses. However, the patterns can be complex and the responses may not be quantitative at any moment of time through stress development, possibly reflecting combinations of immediate and systemic stress responses, and time-lags between stress and onset of emission. More experimental work is needed to quantify the time-kinetics of emission elicitation by various biotic stresses, separate the contributions of immediate and systemically induced emissions, and also address the degree of silencing and priming of emissions.

## Conflict of Interest Statement

The authors declare that the research was conducted in the absence of any commercial or financial relationships that could be construed as a potential conflict of interest.
